# A concise guide to essential R packages for analyses of DNA, RNA, and proteins

**DOI:** 10.1016/j.mocell.2024.100120

**Published:** 2024-10-05

**Authors:** Eng Wee Chua, Der Jiun Ooi, Nor Azlan Nor Muhammad

**Affiliations:** 1 Centre for Drug and Herbal Development, Faculty of Pharmacy, Universiti Kebangsaan Malaysia, 50300 Kuala Lumpur, Malaysia; 2 Department of Preclinical Sciences, Faculty of Dentistry, MAHSA University, 42610 Jenjarom, Selangor, Malaysia; 3 Institute of Systems Biology (INBIOSIS), Universiti Kebangsaan Malaysia, 43600 Bangi, Selangor, Malaysia

**Keywords:** Genomics, Proteomics, R package, Transcriptomics

## Abstract

R is widely regarded as unrivaled by other high-level programming languages for its statistical functions. The popularity of R as a statistical language has led many to overlook its applications outside the statistical realm. In this brief review, we present a list of R packages for supporting projects that entail analyses of DNA, RNA, and proteins. These R packages span the gamut of important molecular techniques, from routine quantitative polymerase chain reaction (qPCR) and Western blotting to high-throughput sequencing and proteomics generating very large datasets. The text-mining power of R can also be harnessed to facilitate literature reviews and predict future research trends and avenues. We encourage researchers to make full use of R in their work, given the versatility of the language, as well as its straightforward syntax which eases the initial learning curve.

## INTRODUCTION

R is popularly known as a statistical language from which a large repertoire of essential statistical tools is derived (R Core Team, 2021). However, the popularity of R in the statistical space may also be its downside. In broader settings of data processing and analysis, R is overshadowed by Python, a related and arguably more versatile language (Carbonnelle, 2024). Also, to some users, especially nonbioinformaticians such as molecular biologists, navigating the unfamiliar command-line interface to execute R “packages” and functions can be a daunting learning curve.

**Fig. 1:**
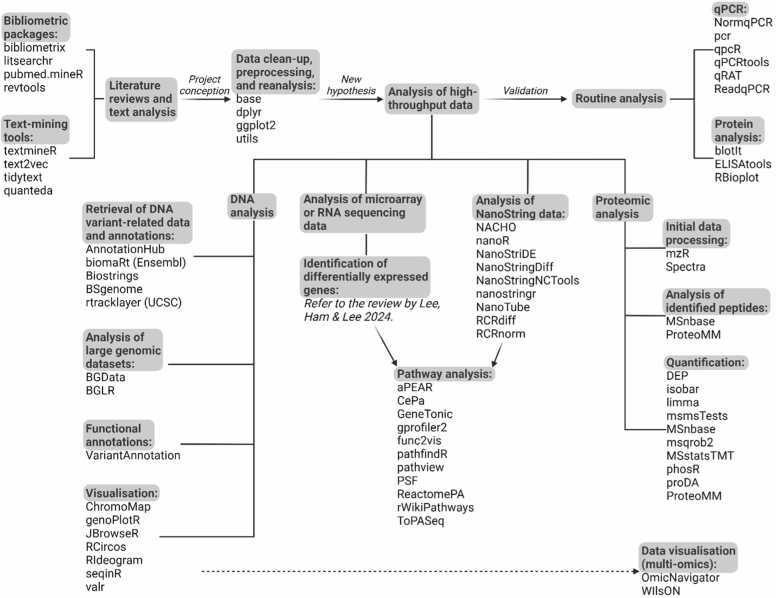
The applications of R packages in molecular biology, ranging from routine analysis to large-scale processing of genomic, transcriptomic, and proteomic data.

In this work, we aim to highlight the utility of R outside the statistical realm, by presenting a nonexhaustive list of useful R packages for supporting projects that entail analyses of DNA, RNA, and proteins (Fig. 1; Table 1). We have used some of these R tools to tackle tasks of varying complexity, ranging from routine analysis to more sophisticated large-scale processing of nucleic acid sequencing data. Being a high-level programming language, R has a syntax that closely resembles natural language; overall, the intuitive structure of R code, coupled with the abundance of learning resources available and the beginner-friendly interface of RStudio for executing R functions, considerably reduces the difficulty of learning the language.

**Table 1:** R packages for supporting literature reviews, data cleaning, and analyses of DNA, RNA, and proteins, along with their uses and strengths

R package	Use	Remarks	Website
Literature reviews—bibliometric tools (the “specialists”)			
bibliometrix	Bibliometric and scientometric analyses	It curates an armamentarium of bibliometric tools; but of relevance is its ability to pinpoint relationships between keywords or any specified type of text and build a *conceptual structure map*, revealing meaningful trends.	https://www.bibliometrix.org/home/
litsearchr	Systematic reviews	It is used primarily to enhance literature search strategies, including identification of optimal keywords and quality assessment of search results.	https://elizagrames.github.io/litsearchr/
pubmed.mineR	Analysis of PubMed abstracts	It is specifically designed to analyze abstracts from the PubMed database, with an extensive range of text-mining functionalities such as text extraction or abstract searches based on specified query terms or arguments. An interesting feature of pubmed.mineR is that the search and data retrieval can also be directed by gene symbols.	https://CRAN.R-project.org/package=pubmed.mineR
revtools	Systematic reviews	It can expedite the process of screening and selecting relevant articles for systematic reviews.	https://cran.r-project.org/package=revtools
Literature reviews—text-mining algorithms (the “generalists”)			
textmineR	These are “general-purpose” text-miners with varied functionalities and not designed to handle the citation records of literature databases. A major difference between them is whether they convert input text into analyzable units or numerical data, ie, tokenization (tidytext and quanteda) vs vectorization (textmineR and text2vec).	Both packages can perform the classic tasks in text analysis, ie, topic modeling which reveals the *patterns* underlying given text, word embedding based on flanking context, and document clustering by computed similarity. TextmineR has better compatibility with other R packages, such as tidytext.	https://cran.r-project.org/package=textmineR
text2vec			https://text2vec.org/
tidytext		Both packages can perform sentiment analysis based on predefined sets of negative and positive words. Additional functionalities include topic modeling and text data visualization. It is worth noting that tidytext abides by the *tidy text* principles and depends on packages from tidyverse.	https://www.tidytextmining.com/
quanteda			https://quanteda.io/
Data preprocessing and cleaning			
base	*Inbuilt R package*	R base functions that underpin the fundamental operations of R, such as mathematical computations and basic data analysis.	Not applicable
dplyr	Data manipulation	This package is part of the immensely popular tidyverse and provides an assortment of functions for manipulating data frames, such as delimited text files that store large variant lists. Key dplyr functions include mutate(), select(), filter(), summarize(), and arrange().	https://dplyr.tidyverse.org/
ggplot2	Data visualization	It can create a variety of graphics, such as box plots, bar charts, and line graphs.	https://ggplot2.tidyverse.org/
utils	*Inbuilt R package*	Housed in this package is a collection of R “utility” functions, such as those for importing, reading, and exporting datasets.	Not applicable
Retrieval and analysis of DNA data			
AnnotationHub	Retrieval and analysis of DNA data	This package is a one-stop “hub” for a repository of DNA-related resources, including UCSC, Ensembl, dbSNP, KEGG, ENCODE, GENCODE, and NCBI.	https://bioconductor.org/packages/AnnotationHub/
biomaRt	Retrieval and analysis of DNA data	By circumventing network bottlenecks, it enables quick access to Ensembl data, including genes, single-nucleotide polymorphisms, and regulatory features. This package can also query non-Ensembl databases.	https://bioconductor.org/packages/biomaRt/
Biostrings	Analysis of DNA data	This package is tailored for sequence-centric analyses, such as DNA sequence extraction, editing, and alignment.	https://bioconductor.org/packages/Biostrings/
BSgenome	Retrieval of reference genomes	This package complements Biostrings. It provides the reference genomes required for analyses performed by Biostrings.	https://bioconductor.org/packages/BSgenome/
rtracklayer	Database access and visualization	It obtains genomic annotations, visually constructed into *tracks*, from browsers such as UCSC.	https://bioconductor.org/packages/rtracklayer/
Handling of big data			
BGData	Used in tandem for analysis of very large genomic datasets	A typical pipeline consists of initial data clean-up and association analysis by BGData, eliminating ineligible subjects and prioritizing DNA variants, and final genomic regression by BGLR, which estimates the effects of genotypes on phenotypes.	https://cran.r-project.org/package=BGData
BGLR			https://cran.r-project.org/package=BGLR
Annotation of DNA variants			
VariantAnnotation	Assessing the functional impact of DNA variants	It reads variant lists in the Variant Call Format (VCF), annotates amino acid changes, and assesses functional relevance. It can also be used to filter VCF files based on specified criteria.	https://bioconductor.org/packages/VariantAnnotation/
Visualization of DNA data			
ChromoMap	Visualization of chromosomes or chromosomal regions	It maps chromosome features (such as genes and single-nucleotide polymorphisms) and visualizes feature-associated data (such as multiomics data).	https://lakshay-anand.github.io/chromoMap/index.html
genoPlotR	Visualization of gene and genome maps	It can easily produce publication-grade graphics of gene and genome maps compared to alternatives such as Artemis, ACT, and mauve.	https://genoplotr.r-forge.r-project.org/
JBrowseR	Accessing JBrowse from R	It provides a simple and clean interface to JBrowse 2 for R users.	https://gmod.org/JBrowseR/
RCircos	Generation of Circos 2D images	It provides a simple and flexible way to generate Circos 2D track plot images for genomic data visualization.	https://cran.r-project.org/package=RCircos
RIdeogram	Visualization of genomic data	It draws SVG graphics to visualize and map genome-wide data on ideograms.	https://github.com/TickingClock1992/RIdeogram
seqinR	Retrieval and analysis of biological sequences	It performs exploratory data analysis and visualizes biological sequences (DNA and protein).	https://seqinr.r-forge.r-project.org/
valr	Data manipulation	It reads and manipulates genome intervals and signals, like the BEDtools suite.	https://rnabioco.github.io/valr/
Transcriptomic analysis with NanoString			
NACHO	These packages handle transcriptomic data generated by the NanoString platform. They can all be used to normalize NanoString counts and differ chiefly in the functionalities they provide for prenormalization or postnormalization data processing and analysis, as well as the methods used in normalizing data. For a detailed discussion, readers are referred to the review by Chilimoniuk et al. (2024). Some of the packages listed by Chilimoniuk et al. (2024) have been removed from the CRAN repository and are thus not included here.	With an easy-to-navigate Shiny interface, it enables users to comprehensively assess data quality and visualize a variety of quality metrics, such as binding intensity and principal components.	https://github.com/mcanouil/NACHO
nanoR		Prenormalization functionalities offered are background correction and quality control. It also carries out differential expression analysis.	https://github.com/KevinMenden/nanoR
NanoStriDE		It is run as a web application to pinpoint differentially expressed genes from normalized data.	http://nanostride.soe.ucsc.edu/
NanoStringDiff		This package enables differential expression analysis postnormalization.	https://bioconductor.org/packages/NanoStringDiff/
NanoStringNCTools		It can perform prenormalization quality assessments of count data.	https://bioconductor.org/packages/NanoStringNCTools/
nanostringr		This package can improve data quality via its prenormalization functionalities for quality control and batch effect correction.	https://cran.r-project.org/package=nanostringr
NanoTube		This package provides the complete set of NanoString-related tools, ie, quality control, normalization, identification of differentially expressed genes, pathway analysis, and data visualization.	https://bioconductor.org/packages/NanoTube/
RCRdiff		These are related packages. RCRnorm was specially devised to address the limitations of data normalization approaches. It performs random-coefficient hierarchical regression, which accounts for different *levels* of variability that stem from probes, genes, and samples, yielding better-normalized data than older methods. A notable feature of RCRnorm is its assumption that the expression of reference genes does not stay perfectly constant across samples and thus should be corrected. RCRdiff is a follow-up package that streamlines the workflow by enabling determination of differentially expressed genes directly from raw count data.	https://github.com/canx2021/RCRdiff
RCRnorm			https://cran.r-project.org/package=RCRnorm
Pathway analysis			
aPEAR	Visualization of pathway enrichment networks	It leverages similarities between the pathway gene sets and represents them as a network of interconnected clusters.	https://gitlab.com/vugene/aPEAR
GeneTonic	Pathway visualization	This package runs a Shiny application that enables integrated exploration of differentially expressed genes and enriched pathways already identified by other tools. A variety of graphics can be generated, such as enrichment maps and heatmaps.	https://federicomarini.github.io/GeneTonic/
gprofiler2	Gene list functional enrichment	It performs over-representation analyses to identify functionally relevant pathways and outputs the results as publication-quality figures.	https://cran.r-project.org/package=gprofiler2
func2vis	Visualization of enriched gene ontology terms	It cleans up enriched gene ontology terms using ConsensusPathDB.	https://cran.r-project.org/package=func2vis
pathfindR	Active subnetwork-oriented enrichment analysis	This R package performs iterations of the following unique workflow for pathway analysis: (1) searches guided by input gene lists to pinpoint active subnetworks, demarcated by protein-protein interactions. (2) filtering of subnetworks based on the number of significantly altered genes. (3) identification of significantly enriched pathways (or terms) within those subnetworks.	https://egeulgen.github.io/pathfindR/
pathview	Pathway enrichment analysis based on primary databases	These packages enable pathway enrichment analyses based on, in descending order of popularity, the KEGG, Reactome, or WikiPathways database (Mubeen et al., 2019). Pathview can be run locally or via a web server (https://pathview.uncc.edu/) to map user-supplied gene or compound lists onto KEGG pathways. ReactomePA pinpoints *over-represented* or *enriched* Reactome pathways within given gene sets. Likewise, rWikiPathways identifies *enriched* pathways from the WikiPathways database.	https://bioconductor.org/packages/pathview/
ReactomePA			https://bioconductor.org/packages/ReactomePA/
rWikiPathways			https://bioconductor.org/packages/rWikiPathways/
CePa	Topology-based pathway analysis	These packages perform topology-based analyses, where the significance of a gene is determined by its location within a pathway and how it interacts with other genes in the same pathway. CePa builds on oft-used workflows for identifying over-represented or enriched pathways, by adding topology information into the analyses. PSF allows users to edit and enhance existing pathways from databases such as KEGG. ToPASeq provides a variety of methods for analyzing pathway topology and layouts.	https://cran.r-project.org/package=CePa
PSF			https://github.com/hakobyansiras/psf
ToPASeq			https://github.com/lgeistlinger/ToPASeq
Proteomic analysis—data preparation			
mzR	Accessing common file formats and parsing mass spectrometry data	This package provides a platform for importing, accessing, and manipulating mass spectrometry data. It integrates easily with other R packages, making it an essential component of a comprehensive pipeline for mass spectrometry data analysis.	https://bioconductor.org/packages/mzR/
Spectra		This package also offers a comprehensive set of tools for manipulating, analyzing, and visualizing mass spectrometry data, as well as integrability with more complex analysis pipelines for advanced workflows.	https://bioconductor.org/packages/Spectra/
Proteomic analysis—visualization			
MSnbase	Mass spectrometry data processing and analysis	This tool is essential for inspecting spectra, analyzing chromatograms, and comparing quantitative data. Its visualization functions help create clear and informative representations of proteomic data.	https://bioconductor.org/packages/MSnbase/
ProteoMM	Model-based peptide-level differential expression analysis of single or multiple datasets	This package is designed for mixed-effects modeling of mass spectrometry proteomics data. While its visualization capabilities focus mainly on model diagnostics, it is a valuable package for applying advanced statistical models in proteomics research.	https://bioconductor.org/packages/ProteoMM/
Proteomic analysis—quantification			
DEP	Label-free quantification (LFQ) of proteomics data	This package provides a comprehensive workflow for analyzing LFQ data, including normalization, imputation, differential expression analysis, and visualization. It can be integrated with other bioinformatics tools for downstream analysis, such as pathway enrichment and protein interaction networks.	https://bioconductor.org/packages/DEP/
MSnbase		The package offers flexible options for customizing LFQ workflows based on specific experimental designs and data characteristics. It is well-documented, but beginners in LFQ analysis and Bioconductor might need some time to learn it.	https://bioconductor.org/packages/MSnbase/
msqrob2		This specialized package provides advanced statistical techniques for analyzing LFQ proteomics data, thereby enhancing the reliability and sensitivity of differential expression analysis. It is ideal for complex experimental designs and large-scale studies but may require significant computational resources.	https://bioconductor.org/packages/msqrob2/
proDA		This R package is designed for LFQ proteomics data analysis. It uses linear models and empirical Bayes methods to reduce variability and improve accuracy in differential expression analysis. Designed to be user-friendly, it simplifies the analysis process and offers clear functions for statistical modeling and result interpretation.	https://bioconductor.org/packages/proDA/
msmsTests		This package provides tools for statistical testing of mass spectrometry data, specifically quantitative analysis in LFQ proteomics. It supports hypothesis testing to identify differentially expressed proteins across different experimental conditions.	https://bioconductor.org/packages/msmsTests/
isobar	Analysis and quantitation of isobarically tagged mass spectrometry proteomics data	This package focuses on the analysis of quantitative data from mass spectrometry experiments that use isobaric tagging (eg, tandem mass tag (TMT), iTRAQ). It also provides basic visualization tools to aid in the interpretation of labeled quantification data.	https://bioconductor.org/packages/isobar/
limma	High-throughput data analysis	Originally developed for microarray data analysis, this package has been adapted for various high-throughput omics data. It effectively handles labeled quantification data by accommodating different labeling strategies such as isotopic labeling and isobaric tagging into its analysis framework.	https://bioconductor.org/packages/limma/
MSnbase	Mass spectrometry data processing and analysis	It is a versatile tool for the manipulating, processing, and visualizing mass spectrometry and proteomics data. It supports a wide range of data types, from raw experimental data to processed quantitative and annotated datasets.	https://bioconductor.org/packages/MSnbase/
MSstatsTMT	Relative quantification of proteins and peptides in mass spectrometry-based proteomics	This package is designed specifically for the analysis of TMT proteomics data. It provides a streamlined workflow for quantitative analysis of isobaric labeling experiments.	https://bioconductor.org/packages/MSstatsTMT/
PhosR	Analysis of phosphoproteomic data	This specialized package focuses on analyzing phosphoproteomics data, particularly phosphorylation site quantification and functional analysis. Beyond quantification, it supports downstream functional analysis including kinase-substrate enrichment, functional annotation, and network analysis.	https://bioconductor.org/packages/PhosR/
ProteoMM	Model-based, peptide-level differential expression analysis of single or multiple datasets	It is a powerful tool for applying mixed-effects models to proteomics data, significantly enhancing the robustness of quantitative analysis in complex experimental designs.	https://bioconductor.org/packages/ProteoMM/
Multiomic analysis			
OmicNavigator	Data exploration and visualization	Both packages run web-based applications for visualization and analysis of omics data, such as those generated from RNA sequencing and measurements of protein abundance. An interesting function of OmicNavigator is that it allows users to create and store study-specific R packages for future viewing within the application and for data sharing.	https://github.com/abbvie-external/OmicNavigator
WIlsON			https://github.com/loosolab/wilson/
Analysis of quantitative polymerase chain reaction (qPCR) data			
NormqPCR	These R packages are used for processing and analyzing qPCR data. They differ mainly in the range of tools they offer, where some have fairly limited functionalities.	It aids the selection of optimal, or *stably expressed*, reference genes, as well as data normalization and relative quantification. Two methods, geNorm and NormFinder, are used to determine the suitability of reference genes.	https://bioconductor.org/packages/NormqPCR/
pcr		It can compute amplification efficiency and quantify gene expression using the ΔΔC_q_ or standard curve method.	https://cran.r-project.org/package=pcr
qpcR		It overcomes the inaccuracies of methods that ignore sample-to-sample variability when estimating amplification efficiency. By overlaying a sigmoidal model onto the fluorescence-vs-cycle curve, it estimates sample-specific amplification efficiency and quantification cycles from the fitted model, not unlike how gene-level amplification efficiency is determined from a dilution series. The package also includes conventional methods for computing amplification efficiency.	https://cran.r-project.org/package=qpcR
qPCRtools		It can calculate the amount of RNA for reverse transcription, assess amplification efficiency, and compute gene expression levels. Users can select one of the three approaches available for gene quantification, ie, the ΔΔC_q_, standard curve, or reference-independent method, based on the amplification efficiency of the reference and target genes.	https://cran.r-project.org/package=qPCRtools
qRAT		With raw C_q_ values stored as .csv or .txt files, this R package can perform the following tasks: (1) quality assessment, eliminating outliers; (2) relative quantification, using reference-normalized C_q_ values; (3) inter-run calibration, allowing cross-plate comparisons; and (4) data visualization.	https://qrat.shinyapps.io/qrat/
ReadqPCR		This is a prerequisite package for NormqPCR. It reads qPCR data, ie, C_q_ values, into R.	https://bioconductor.org/packages/ReadqPCR/
Analysis of data obtained from Western blotting or enzyme-linked immunosorbent assay (ELISA)			
blotIt	Data preprocessing	It is useful for rescaling Western blots obtained from separate experiments, which would be otherwise unsuitable for comparison due to inter-run variation.	https://github.com/JetiLab/blotIt
ELISAtools	Analysis of ELISA data	It computes protein concentrations from standard curves of optical density readings, while simultaneously correcting for batch effects.	https://cran.r-project.org/package=ELISAtools
RBioplot	Statistical analysis and data visualization	This package can read .csv files containing values for relative protein abundance, carry out statistical analysis, and generate graphics, ie, histograms, heatmaps, and join-point curves.	https://jzhangc.shinyapps.io/rbioplot_shiny/

## R PACKAGES FOR INITIAL DATA ANALYSIS AND PROJECT CONCEPTION

### R Syntax and Core Packages

R code is constructed in a right-branching manner, akin to English grammar dictating the placement of nouns after verbs. For instance, to implement the *library()* function, which loads a package or tool into the R environment before it can be executed, the code should be written as *library(package)*. Important inbuilt R packages include utils with functions such as *read.delim()* for importing delimited data into R; *head()* and *tail()* for viewing segments of datasets; and *write.table()* for exporting processed data. The R base package comprises an assemblage of functions for basic operations in R such as mathematical computations and data manipulation. For example, the *grep()* and *grepl()* functions can be used to find pieces of text matching a given pattern. Another notable feature of R syntax is the use of the pipe operator, *%>%*, to link a series of functions performed consecutively on the same object, and this contributes to the overall conciseness of R code. For R beginners, a variety of free online courses are available on Codecademy (https://www.codecademy.com/), Udemy (https://www.udemy.com/), DataCamp (https://www.datacamp.com/), Coursera (https://www.coursera.org/), and many other similar platforms. These courses cover the fundamentals of R and provide practice problems with mixed levels of difficulty.

### Basic Data Processing

Tidyverse (Wickham et al., 2019) comprises an assortment of packages for efficient processing or cleaning up of large volumes of data to generate input files for further analysis; or to enable pooled reanalysis of existing datasets, yielding new hypotheses or supporting evidence for project proposals. An oft-used tidyverse package is dplyr, which provides handy functions such as *arrange()* for sorting tables by column values; *filter()* for retaining rows according to certain criteria; *group_by()* for aggregating values from multiple rows; and *mutate()* for creating new columns. Another “must-have” tidyverse package is ggplot2 for data visualization. The code for converting gene-level read counts produced by RNA sequencing into a Gene Set Enrichment Analysis (GSEA)-compatible format can be found in Supplementary Materials.

### Literature Searches and Text Analysis

The repository of scientific literature is growing at a rate far exceeding researchers’ ability to keep pace. R can facilitate literature reviews through specialized packages for bibliometric analysis, ie, bibliometrix (Aria and Cuccurullo, 2017), litsearchr (Grames et al., 2019), pubmed.mineR (Rani et al., 2015), and revtools (Westgate, 2019); and text-mining algorithms designed to distil meaningful patterns from words and characters, ie, tidytext (Silge and Robinson, 2017), quanteda (Benoit et al., 2018), text2vec (Selivanov et al., 2020), and textmineR (Jones, 2023).

In general, bibliometric R packages are more efficient at processing the findings of literature searches, as their built-in functions can complete the task with merely a few lines of code. For instance, bibliometrix aids systematic reviews through functionalities that automatically recognize the citation records from different databases, extract text, and assess word co-occurrences, constructing a “conceptual structure map” that reveals trending research areas. The more general-purpose text-mining tools, while less straightforward, offer room for customization and insights into the mechanics of the analysis. For instance, to pinpoint the most frequently appearing words within the results of a PubMed search, tidytext first renders input text into discrete data units; then it removes stop words, computes word frequencies, and outputs a ranked list of words. However, a significant drawback of tidytext is its inability to directly read database-specific output files, such as nbib for PubMed, into R as analysis-ready data frames.

## R PACKAGES FOR THE ANALYSES OF DNA, RNA, AND PROTEINS

### Genomic and Transcriptomic Analyses

R can also retrieve genomic data and annotations from online databases, bypassing web interfaces to expedite high-volume data transfer. BiomaRt fetches data from the Ensembl database for DNA variant-oriented analysis (Durinck et al., 2009); rtracklayer enables access to annotations from the UCSC Genome Browser (Lawrence et al., 2009). BSgenome and Biostrings are usually used in tandem for genome-centric analyses (Pagès, 2024). AnnotationHub is a one-stop “hub” for a large variety of DNA databases (Morgan and Shepherd, 2024; Table 1). BGData contains R packages for handling large genomic datasets (Grueneberg and de Los Campos, 2019), alongside BGLR which performs the final regression analysis to identify genotype-phenotype associations (Pérez and de los Campos, 2014). VariantAnnotation (Obenchain et al., 2014) is useful for ascertaining the functional impact of novel DNA variants. R packages used in visualizing genomic data include seqinR (Charif et al., 2005), genoPlotR (Guy et al., 2010), RCircos (Zhang et al., 2013), valr (Riemondy et al., 2017), RIdeogram (Hao et al., 2020), JBrowseR (Hershberg et al., 2021), and ChromoMap (Anand and Rodriguez Lopez, 2022). Combined with base R functions, all these tools are remarkably effective. For instance, we have used biomaRt to gauge the “editability” of known human single-nucleotide polymorphisms by retrieving their flanking sequences from Ensembl and examining for the presence of clustered regularly interspaced short palindromic repeats (CRISPR)-binding motifs, such as “GG” (Supplementary Materials).

R is also widely used in processing data obtained from RNA sequencing or microarray analysis, which entail similar bioinformatics workflows. Readers are referred to the review by Lee et al. (2024) for a detailed list of R packages used in identifying differentially expressed genes. Likewise, NanoString has also earned a dedicated set of R-based tools (Chilimoniuk et al., 2024), such as nanoR (https://github.com/KevinMenden/nanoR), NanoStriDE (Brumbaugh et al., 2011), nanostringr (Talhouk et al., 2016), NanoStringDiff (Wang et al., 2016), RCRnorm (Jia et al., 2019), NACHO (Canouil et al., 2020), RCRdiff (Xu et al., 2022), NanoTube (Class et al., 2023), and NanoStringNCTools (Aboyoun and Ortogero, 2024). Transcriptomic analyses conclude with the determination of enriched pathways, using R packages such as func2vis (https://cran.r-project.org/web/packages/func2vis/index.html), CePa (Gu and Wang, 2013), pathview (Luo and Brouwer, 2013), ToPASeq (Ihnatova and Budinska, 2015), ReactomePA (Yu and He, 2016), rWikiPathways (Slenter et al., 2018), pathfindR (Ulgen et al., 2019), gprofiler2 (Kolberg et al., 2020), GeneTonic (Marini et al., 2021), PSF (Hakobyan et al., 2023), and aPEAR (Kerseviciute and Gordevicius, 2023). Increased cross-omics integration has led to the development of R-based tools for visualizing multiomics data, such as OmicNavigator (Ernst et al., 2024) and WIlsON (Schultheis et al., 2019).

### Proteomic Analysis

Proteomic analysis involves the large-scale study of proteins, where the raw data originate primarily from mass spectrometry. Sophisticated analysis is required to extract meaningful biological information from these data, including chromatograms and fragment ion spectra. Analysis of proteomic data is a multistep process involving various computational tools. While specialized software dominates the space of peptide identification, R excels in data manipulation, statistical analysis, and data visualization.

For data analysis and visualization, R offers robust capabilities through packages like mzR (Chambers et al., 2012) and Spectra (Rainer et al., 2022), which facilitate the initial handling of raw mass spectrometry data. The mzR package allows access to mass spectrometry data in various open formats, such as mzXML, mzData, mzML, and netCDF, while Spectra enables initial data exploration and visualization. The actual identification of peptides is typically handled by third-party software tools, including Sequest (Eng et al., 1994), Mascot (Perkins et al., 1999), PEAKS (Zhang et al., 2012), MaxQuant (Tyanova et al., 2016), Comet (Schweppe et al., 2020), and Proteome Discoverer (Orsburn, 2021). Then, identified peptides can be imported into R for statistical analysis and visualization using packages such as MSnbase (Gatto et al., 2020) and ProteoMM (Karpievitch et al., 2024).

For relative and absolute quantification of protein expression levels across samples, either label-free or labeled approaches are used. Key R packages for label-free quantification include DEP (Zhang et al., 2018), MSnbase (Gatto et al., 2020), msqrob2 (Sticker et al., 2020), proDA (Ahlmann-Eltze, 2024), and msmsTests (Gregori et al., 2024). Labeled quantification involves chemical or metabolic modifications of peptides or proteins, enabling more accurate relative quantification of protein levels across samples. Although R does not have dedicated packages for labeled quantification, other multifunction R packages can be used for this purpose, including isobar (Breitwieser et al., 2011), limma (Ritchie et al., 2015), MSnbase (Gatto et al., 2020), MSstatsTMT (Huang et al., 2020), PhosR (Kim et al., 2021), proDA (Ahlmann-Eltze, 2024), and ProteoMM (Karpievitch et al., 2024).

### Routine Data Analysis

A variety of R packages are available for preprocessing and analyzing quantitative polymerase chain reaction (PCR) data, such as qpcR (Ritz and Spiess, 2008), ReadqPCR (Perkins et al., 2012), NormqPCR (Perkins et al., 2012), pcr (Ahmed and Kim, 2018), qPCRtools (Li et al., 2022), and qRAT (Flatschacher et al., 2022). The functionalities these packages offer include evaluating primer efficiency, correcting C_q_ values for inter-run variation, computing relative gene expression levels, and selecting optimal reference genes. qRAT, built from Shiny, is available both as a locally run package for skilled R users and as a web-based application with an easy-to-use interface (https://qrat.shinyapps.io/qrat/).

The intensities of chemiluminescent signals can vary considerably between Western blots; blotIt is a useful R package for combining Western blots from different experiments, as it performs calibration and adjustments to eliminate inter-blot variation in chemiluminescence measurements (Kemmer et al., 2022). Similarly, batch-to-batch variation in enzyme-linked immunosorbent assays (ELISA) can be overcome by ELISAtools (Feng et al., 2019). Last, RBioplot can be used to automate statistical analysis and visualization of data produced by both quantitative PCR and protein measurements (Zhang and Storey, 2016).

## CONCLUDING REMARKS

In this brief review, we have presented a list of R packages that span the gamut of key molecular analyses involving DNA, RNA, and proteins. We have shown that in terms of versatility, R is not inferior to other high-level programming languages. An oft-cited barrier to the adoption of R is its inaccessible command-line environment; but this is a misconception that should be debunked, considering the straightforward syntax of R, and that should not deter researchers from making full use of R-based tools in their work. By effectively combining these tools, researchers can gain valuable insights into gene and protein functions and disease mechanisms.

## ORCID

Eng Wee Chua: https://orcid.org/0000-0002-1612-007X

Der Jiun Ooi: https://orcid.org/0000-0002-4896-2351

Nor Azlan Nor Muhammad: https://orcid.org/0000-0003-4401-0385

## Author Contributions

**Eng Wee Chua:** Writing—review and editing, Writing—original draft, Conceptualization. **Der Jiun Ooi:** Writing—review and editing, Writing—original draft. **Nor Azlan Nor Muhammad:** Writing—review and editing.

## Declaration of Competing Interests

The authors declare that they have no known competing financial interests or personal relationships that could have appeared to influence the work reported in this paper.
