# Supplementary material for: A concise guide to essential R packages for analyses of DNA, RNA, and proteins
Source: Mol Cells. 2024 Oct 5;47(11):100120. doi: 10.1016/j.mocell.2024.100120 (PMC11541695; doi:10.1016/j.mocell.2024.100120)
Supplement: Supplementary material [file mmc1.docx]

**A Concise Guide to Essential R Packages for Analyses of DNA, RNA, and Proteins – Supplementary Materials**

**CASE STUDY 1: CONVERSING READ COUNTS INTO GSEA-COMPATIBLE FORMAT**

gsea <- data.frame(nreadcount) %>%

tibble::rownames_to_column("Name") %>%

mutate(Description = "NA", .after = Name)

header <- list(

c("#1.2", nrow(gsea)),

c("", ncol(gsea) - 2)

)

write.table(header, file = "path/gsea.gct",

quote = FALSE,

sep = "\t",

col.names = FALSE,

row.names = FALSE)

write.table(gsea, file = "path/gsea.gct",

quote = FALSE,

sep = "\t",

col.names = TRUE,

row.names = FALSE,

append = TRUE)

**CASE STUDY 2: CHECKING THE EDITABILITY OF SINGLE-NUCLEOTIDE POLYMORPHISMS**

BiocManager::install("biomaRt") #Make sure you have the latest version of R (>=4.4).

library(biomaRt)

library(tidyverse)

#To interrogate Ensembl SNP databases, i.e. hsapiens_snp, hsapiens_snp_som, hsapiens_structva, and hsapiens_structvar_som:

ensembl_snps <- useEnsembl(biomart = "snps", dataset = "hsapiens_snp")

#Alternatively if you want to know what's available:

listEnsembl()

ensembl_snps <- useEnsembl(biomart = "snps")

listDatasets(ensembl_snps)

searchDatasets(mart = ensembl_snps, pattern = "hsapiens")

ensembl_snps <- useDataset(dataset = "hsapiens_snp", mart = ensembl_snps)

listAttributes(ensembl_snps) #The desired output

listFilters(ensembl_snps) #The filters that will be applied to the dataset

#To get flanking sequences for SNPs (source of the code: https://support.bioconductor.org/p/89688/):

target_snps <- c("rs1065852", "rs113993960", "rs3808607") #Examples of SNPs

flanking_sequences <- getBM(

attributes = c("refsnp_id", "snp"),

filters = c("snp_filter", "upstream_flank", "downstream_flank"),

checkFilters = FALSE,

values = list(target_snps, 20, 20),

mart = ensembl_snps,

bmHeader = TRUE)

#To split the upstream and downstream flanking sequences from variants:

flanking_sequences_split <- flanking_sequences %>% separate_wider_delim(

"Variant sequences",

"%",

names = c("upstream_seq", "SNP", "downstream_seq")

)

#To check if a SNP can be edited by CRISPR:

flanking_sequences_split$CRISPR <- grepl("GG", flanking_sequences_split$upstream_seq, ignore.case = TRUE) | grepl("GG", flanking_sequences_split$downstream_seq, ignore.case = TRUE)

summary(flanking_sequences_split$CRISPR)

#To get a list of mtDNA variants from the Ensembl database:

mtDNA_variants <- getBM(

attributes = c("refsnp_id", "chr_name", "chrom_start", "snp"),

filters = "chr_name",

values = "MT",

mart = ensembl_snps,

bmHeader = TRUE)

**CASE STUDY 3: DETERMINING THE CURRENT RESEARCH TRENDS**

library(tidytext)

library(dplyr)

#To read Pubmed search results into R:

pubmed_search <- read.delim(

"path/csv-CRISPRANDc-set.csv",

header = TRUE,

sep = ",")

#Alternatively, the following line of code can be used to load an .nbib file into R.

#revtools::read_bibliography("path/pubmed-crisprandc-set.nbib")

#To build a tibble dataframe from the list of titles:

text_df <- tibble(

line = 1:nrow(pubmed_search),

text = pubmed_search$Title)

#To covert the titles into tidytext format (one word per row):

text_tidied <- text_df %>% unnest_tokens(word, text)

#To remove stop words:

data(stop_words)

text_tidied <- text_tidied %>% anti_join(stop_words)

#To remove keywords used in the search:

text_filtered <- text_tidied %>% filter(

word != "crispr",

word != "cas9",

word != "cancer",

word != "gene",

word != "editing",

word != "tumor")

text_filtered %>% count(word, sort = TRUE)
